# The Impact of Plant-Derived Foods and Medicines on the Lung–Gut Axis and Microbiota Cross-Talk

**DOI:** 10.3390/molecules31152699

**Published:** 2026-08-03

**Authors:** Sarocha Vivatvakin, Duangporn Werawatganon

**Affiliations:** Center of Excellence in Alternative and Complementary Medicine for Gastrointestinal and Liver Diseases, Department of Physiology, Faculty of Medicine, Chulalongkorn University, Bangkok 10330, Thailand; sarocha.v@chula.ac.th

**Keywords:** airway inflammation, asthma, gut-lung axis, lung-gut axis, microbiota, plant-based, polysaccharides, polyphenols, respiratory infections, short-chain fatty acids

## Abstract

The gut–lung axis is one of the bidirectional mucosal communication networks linking intestinal microbiota, microbial metabolites, immune regulation, and respiratory inflammation. This narrative review summarizes mechanistic and translational evidence identified through a structured but non-systematic search. This review focuses on gut-to-lung mechanisms, including immune cell trafficking, epithelial barrier regulation, and the role of gut microbiota and its metabolites. Among these metabolites, short-chain fatty acids (SCFAs), particularly acetate, propionate, and butyrate, are key mediators of interorgan communication and are mainly produced through microbial fermentation of dietary fibers and polysaccharides. Unlike previous reviews, reverse lung-to-gut mechanisms are also discussed, whereby respiratory infection, smoking, and lung injury may disrupt intestinal barrier integrity, alter gut microbiota composition, and promote intestinal immune dysregulation. Among plant-derived interventions, dietary fibers, polysaccharides, and polyphenols appear promising, mainly through SCFA-dependent immune modulation, enrichment of metabolite-producing bacteria, and subsequent suppression of inflammatory pathways. Some compounds may also act through microbiota-independent mechanisms, including direct antioxidant effects, epithelial barrier protection, and inhibition of inflammatory signaling pathways. Overall, SCFA-mediated immune regulation is the best-supported mechanism, whereas lung-to-gut signaling remains less established. Most evidence remains preclinical, highlighting the need for standardized human studies evaluating dose, safety, and clinically meaningful respiratory outcomes.

## 1. Introduction

The gastrointestinal and respiratory tracts are anatomically distinct mucosal surfaces, but they are functionally connected through shared immune and metabolic pathways. Emerging evidence indicates that the microbiota are essential through these pathways in maintaining normal physiological functions of the human organs, whereas dysbiosis of these microbial communities in both the gut and lungs is intimately linked to the pathogenesis of various pulmonary and systemic diseases [1]. There is a bidirectional communication network between these two organs, which is commonly referred to as the gut–lung axis. Unlike the well-known gut–brain or gut-liver axis, data regarding the gut-lung axis are still relatively unclear. In this axis, intestinal microbial communities and microbiota-derived metabolites contribute to mucosal immune regulation, epithelial barrier integrity, and inflammatory responses [2,3]. In contrast, the lung–gut axis is a less well-characterized component of this bidirectional communication network and refers to the reverse direction of the gut–lung axis. The lung-gut axis refers to the effect of respiratory infection and lung injury that may result in alteration of intestinal permeability, gut microbiota composition, and intestinal immune responses. Although these two axes share common mediators, they differ in their initiating organs, the direction of communication, and the strength of the available evidence. However, the mechanisms between these two directions remain incompletely understood, particularly the lung-gut axis. A mechanistic review is therefore needed to provide a clear view of these relationships.

Among microbiota-derived metabolites, short-chain fatty acids (SCFAs) are among the most important mediators of gut–lung communication. SCFAs, mainly acetate, propionate, and butyrate, are produced through bacterial fermentation of non-digestible plant-derived carbohydrates, including dietary fibers [4]. These metabolites provide a mechanistic link between plant-based intake, gut microbial activity, and pulmonary immune regulation. Therefore, plant-derived fibers and related compounds such as polyphenols are not only nutritional substrates, but they might also be potential regulators of microbiota-dependent immune signaling through the gut–lung axis [5]. Therefore, this review was written to analyze existing research findings and clarify the basic mechanisms of gut–lung communication, with additional attention to the less well-characterized lung–gut axis, in order to clarify how bidirectional mucosal crosstalk contributes to respiratory and gastrointestinal health and disease, and how plant-based substances influence these axes.

## 2. Literature Search Strategy and Study Selection

This article was conducted as a narrative review. A structured but non-systematic literature search was performed to identify relevant studies on the gut–lung axis, lung–gut axis, microbiota-mediated immune regulation, plant-derived substances, and respiratory diseases. PubMed/MEDLINE was searched from database was searched between 15th May and 2nd June 2026. Additional relevant articles were identified by screening the reference lists of selected articles and review papers.

The literature search consisted of two complementary components. First, a targeted narrative search was used to identify key mechanistic and conceptual literature related to gut–lung and lung–gut communication. Search terms included combinations of “gut-lung axis”, “lung-gut axis”, “gut microbiota”, “intestinal microbiota”, “lung microbiota”, “dysbiosis”, “short-chain fatty acids”, “microbial metabolites”, “mucosal immunity”, “airway inflammation”, “respiratory infection”, “asthma”, “COPD”, and “acute lung injury.” These articles were used to establish the biological framework of bidirectional gastrointestinal–respiratory communication, including microbial metabolites, epithelial barrier function, immune-cell trafficking, and systemic inflammatory pathways.

Second, a focused PubMed/MEDLINE search was performed to identify studies examining plant-derived substances in relation to microbiota-mediated respiratory disease or gut–lung axis mechanisms. The following search strategy was used (“Gut-Lung Axis”[tiab] OR “gut lung axis”[tiab] OR “gut-lung axis”[tiab] OR “gut microbiota”[tiab] OR “intestinal microbiota”[tiab] OR “gut microbiome”[tiab] OR “intestinal microbiome”[tiab] OR “Microbiota”[MeSH] OR “Gastrointestinal Microbiome”[MeSH] OR “dysbiosis”[tiab]) AND (“phytochemical*”[tiab] OR “polyphenol*”[tiab] OR flavonoid*[tiab] OR “plant-derived”[tiab] OR “plant based”[tiab] OR “plant-based”[tiab] OR “plant extracts”[MeSH] OR “dietary fiber”[tiab] OR fibre[tiab] OR fiber[tiab] OR prebiotic*[tiab] OR “herbal medicine”[tiab] OR “Herbal Medicine”[MeSH] OR “Phytochemicals”[MeSH] OR “Polyphenols”[MeSH] OR “Dietary Fiber”[MeSH]) AND (“respiratory disease*”[tiab] OR asthma[tiab] OR COPD[tiab] OR “chronic obstructive pulmonary disease”[tiab] OR “airway inflammation”[tiab] OR “lung inflammation”[tiab] OR “allergic airway”[tiab] OR “Respiratory Tract Diseases”[MeSH] OR “Asthma”[MeSH] OR “Pulmonary Disease, Chronic Obstructive”[MeSH]). This search strategy identified 521 records for eligibility review.

Eligible publications for the mechanistic gut–lung and lung–gut axis sections included original experimental studies, clinical studies, and relevant review articles addressing at least one of the following topics, including gut or lung microbiota in respiratory disease, gut-derived microbial metabolites, and immune or epithelial mechanisms involved in gut–lung or lung–gut communication. Both human and animal studies were considered because much of the mechanistic evidence in this field is derived from experimental models. For the section on plant-derived modulation of the gut–lung axis, eligible publications included original experimental studies and clinical studies in which a plant-based component such as dietary fibers, polysaccharides, polyphenols, flavonoids, and plant-derived extracts was used as an intervention, and the study provided information on these interventions’ effects on respiratory disease or lung inflammation along with possible underlying mechanisms. Studies were excluded if they were unrelated to microbiota, gut–lung or lung–gut communication, respiratory disease, or plant-derived interventions; focused solely on prebiotics, probiotics, antibiotics, or synthetic non-plant-based interventions; lacked sufficient methodological detail; or had no accessible full text.

## 3. Microbiota

Microbiota refers to the community of living microorganisms including bacteria, yeasts, and viruses that inhabit a specific environment, such as the oral cavity or the gastrointestinal tract. In contrast, the microbiome refers not only to the living organisms themselves, but also to the collective genetic material of these microorganisms, along with their structural components, metabolic products, and the surrounding environmental context [3]. The first descriptions of the human microbiota were recorded between 1670 and 1680 by Antonie van Leeuwenhoek. Using a handcrafted microscope, he discovered five different types of small living organisms in specimens taken from his own mouth, as well as from the oral and fecal samples of others. He referred to these organisms as animalcules, which are now known as bacteria. He also noted that different body sites and varying health conditions were associated with distinct bacterial populations [6].

Human microbiota varies across different organs. The gut is the most well-established organ in terms of microbiota composition. However, other organs also contain microbiota, but in smaller amounts. The oral cavity represents the second largest microbial community in humans. It can be further divided into several sub-sites, including saliva, the tongue, tooth surfaces, gums, buccal mucosa, palate, and subgingival and supragingival plaque. The major bacterial phyla present in the oral cavity are *Firmicutes*, *Proteobacteria*, *Bacteroidetes*, *Actinobacteria*, and *Fusobacteria*. Other organs, including the lungs, skin, and vagina, also contain distinct microbiota [3].

### 3.1. Gut Microbiota

Approximately 10^4^ species of bacterial flora and up to 14 bacterial genera inhabit the human gastrointestinal tract, with more than 10^11^–10^12^ microorganisms per gram of intestinal tissue [2,3,7,8]. Colonization of these bacteria begins early in life, starting soon after birth. The composition of these gut microbiota changes over time in infants [7]. They become similar to those in adulthood after more than one to three years of age, as solid foods are introduced [3,7]. The main children’s microbiota is *Akkermansia muciniphilia*, *Bacteroides*, *Veillonella*, *Clostridium coccoides* spp., and *Clostridium botulinum* spp. The amount of microbiota increases with age [9]. In healthy individuals, approximately 90% of the normal gut flora are anaerobes, while facultative anaerobes and aerobes comprise the remaining portion [8]. The predominant gut microbiota identified in order of numerical importance are *Firmicutes, Bacteroidetes,* and *Actinobacteria.* The lesser-in-number bacterial genera are *Verrucomicrobia, Fusobacteria, Spirochaetes*, *Proteobacteria*, and *Verrucomicrobia* [10]. The number of gut microbiota decreases after more than 70 years of age. There is a decrease in *Bifidobacterium* and an increase in *Clostridium* and *Proteobacteria*. The attenuation of the inflammatory status is related to the decrease in *Bifidobacterium*, an anaerobic species, which plays a role in enhancing the human immune system [11]. Rather than bacteria, other organisms are found in the intestine, including fungi such as *Candida*, *Saccharomyces*, *Malassezia*, and *Cladosporium;* viruses; phages; and archaea [12,13].

Variation in physicochemical factors such as intestinal motility, pH, host secretions including gastric acid, bile, digestive enzymes, and mucus, and the presence of an intact ileocecal valve lead to distinctive bacterial distributions along different segments of the gut. Consequently, only a few bacterial species are present in the upper gastrointestinal tract due to its harsh environment characterized by low pH and its phasic propulsive motor activity [14,15]. In contrast, the lower gastrointestinal tract, particularly the colon, contains a large number of viable bacteria [15]. The small intestine has a shorter transit time and higher bile concentration, while the colon has slower flow rates, more space, and more nutrients, which is more suitable for microbial communities [14]. Furthermore, several factors can influence bacterial colonization, including antibiotic use, pre- or probiotic use, illness, stress, aging, diet, living environment, and lifestyle [16].

#### 3.1.1. Essential Metabolite Production

Normal gut flora perform various essential functions in maintaining physiologic hemostasis [17]. These include generation of some metabolites by microbiota that could not be found in the diet or produced by human cells [18]. One of these metabolites is short-chain fatty acids (SCFAs) such as acetic, propionic, and butyric acids through fermentation of non-digestible dietary fibers, including undigested complex carbohydrates [4]. This process primarily occurs in the ileum and proximal region of the cecum and colon. The products, especially for butyrate, not only become an energy source for the colonic mucosa and peripheral tissue, but also play a role in protective factors for the intestinal barrier [19]. Further benefits of the gut microbiota are vitamin K synthesis, especially in patients with low vitamin K intake; and deconjugation of bile acids for enterohepatic circulation [17]. Bile acids are secreted into the intestinal lumen in conjugated forms, which increase their solubility, but are poorly absorbed by the intestinal mucosa. Therefore, gut microbial enzymes help deconjugate these compounds, facilitating further modification and passive reabsorption [20].

#### 3.1.2. Barrier Integrity Promotion

Moreover, they play a crucial role in promoting barrier integrity and protecting against pathogenic or exogenous microorganisms’ invasion of the mucosa [17]. The normal gut barrier integrity consists of two main barriers, which are the mechanical and immune barriers. The mechanical barrier includes a single layer of intestinal epithelial cells (enterocytes) and mucus. The gut immune barrier functions consist of secreted immunoglobulin A (IgA) and gut-associated lymphoid tissue (GALT), including intraepithelial lymphocytes, Peyer’s plaques, lamina propria, and mesenteric lymph node [7].

The gut microbiota enhances the mechanical barrier of the gut by various mechanisms. They stimulate intestinal epithelial proliferation and differentiation as well as regulate human intestinal epithelial tight-junction proteins, resulting in strengthening of the epithelial barrier, which prevents pathogenic bacterial invasion [17,21]. The gut microbiota acts directly by competition for nutrients and mucosal binding sites in the colon, resulting in preventing the growth of foreign bacteria. Regarding the SCFAs produced by these microbes, these acids decrease the intestinal and fecal pH by influencing colonic water absorption, resulting in inhibiting the growth of the invasive bacteria [7]. The gut microbiota also produces toxic metabolites from proteolytic fermentation in the distal colon and carcinogenic metabolites such as bacteriocins, ammonia, and phenols that also inhibit the pathogenic bacterial growth [22].

#### 3.1.3. Local Immune Modulation

##### Microbe-Associated Molecular Patterns (MAMPs)

Regarding the immune barrier, gut microbiota influences the mucosal immune system with both pro-inflammatory and regulatory effects. The gut microbiota contains lipopolysaccharides (LPSs) and peptidoglycan (PGN), components of the bacterial cell wall. They have the ability to modulate gut immunity [23]. These microbe-associated molecular patterns (MAMPs) are recognized by human cells, which express pattern-recognition receptors (PRRs) such as Toll-like receptors (TLRs) or NOD-like receptors (NLRs), resulting in modulating the host immune response [18]. These MAMPs individually or synergistically stimulate the nuclear factor-kB (NF-κB) effector production, resulting in the generation of inflammatory cytokines including tumor necrosis factor-α (TNF-α), interleukin-1β (IL-1β), and antimicrobial peptides, which act as the defense against exogenous pathogens in response to acute and excessive stimulation. Furthermore, chronic stimulation of the PRRs by PGN can prime the antigen-presenting cells or dendritic cells, leading to increased function of active regulatory T cells (CD4+CD26L+) (Treg). These result in inhibitory cytokine production, including transforming growth factor β (TGF-β) and IL-10, which helps minimize excessive tissue injury from inflammation [23].

##### Short-Chain Fatty Acids (SCFAs)

Again, the production of SCFAs, especially butyrate, has an immunomodulatory effect by interacting with G protein-coupled receptor (GPR) 109A or hydroxycarboxylic acid receptor 2 (HCAR2) and enhancing differentiation of the naïve T cells into Tregs, resulting in an immunosuppressive state [24,25]. Furthermore, it acts directly through acetylation of key gene sites such as Forkhead Box Protein P3 (Foxp3) by inhibiting histone deacetylase (HDAC) activity, which promotes the differentiation and function of Tregs, resulting in suppression of proinflammatory cytokine production [24]. Acetate signals through GPR43 or free fatty acid receptor (FFAR) 2 to regulate neutrophil and innate lymphoid cells 3 (ILC3)–mediated defense against extracellular bacteria and fungi [26]. These gut microbiota, not only bacteria, but fungi including *fumigatus*, are also able to generate SCFAs [27].

##### Other Gut Microbial-Related Metabolites

There are some other gut-derived microbial metabolites that are documented to have immunomodulatory effects, including indole derivatives produced from dietary tryptophan metabolism, niacin, polyamines (PAs) produced from L-arginine metabolism, urolithin A, and organic acids such as pyruvate and lactate [18]. Administration of lactate or pyruvate stimulates C-X3-C motif chemokine receptor 1 (CX3CR1)^+^ monocytes with intact GPR31 to protrude their dendrites into the intestinal lumen to sample for luminal antigen, thereby enhancing immune responses and resistance to *Salmonella* infection [28].

Segmented filamentous bacteria (SFB), including members of the *Bifidobacterium* and *Bacteroides* genus, produce antimicrobial peptides, secretory IgA, and pro-inflammatory cytokines resulting in supporting the immunity of the gut barrier. Apart from bacteria, fungi also have potential benefits in the aspect of intestinal infection. A previous animal model study showed that *C. albicans* increased the level of IL-17A, which resulted in a protective effect against *C. difficile* infection [29]. *Saccharomyces boulardii* also has protective defense against *C. difficile* infection by its secreted protease that digests toxins A and B [30].

Any interventions that alter the balance of the normal flora may result in the overgrowth of endogenous flora or infection of exogenous pathogens. This abnormality can interfere with the circulating lymphocytes, leading to systemic immune dysregulation. Persistent dysbiosis may progress from local to systemic inflammation and outgrowth of opportunistic pathogens [17].

### 3.2. Lung Microbiota

The lungs were once considered a sterile organ; however, advances in novel detection technologies have revealed that they harbor low-biomass bacterial communities [31]. As the lungs have the largest surface area of 50–100 m^2^, they are exposed to more than 7000 L of microbial-filled air daily [31,32]. The gut and lungs are the main organs which expose to the external environment most [24]. Eventually, the respiratory tract comprises 10^3^–10^5^ CFU per gram of lung tissue [33] and more than 600 bacterial species [34].

Lung microbiota begin colonization in the airway since childbirth [35]. Pattaroni et al. demonstrated that a detectable lower airway microbiota is present within 24 h of birth, using aspiration through an endotracheal tube under sterile conditions and contamination control strategies including endotracheal tube flushing, DNA extraction, and PCR negative controls [35]. The main genera of lung microbiota include *Prevotella*, *Veillonella*, and *Streptococcus*, based on the study investigated by 16S rRNA gene sequencing, which is the same in both childhood, from 7 weeks of age, and adulthood [34,35]. Mode of delivery may influence lung microbiota composition, with cesarean section favoring skin-associated taxa such as *Staphylococcus* spp., whereas vaginal delivery is more commonly associated with vaginal microbes, including *Ureaplasma* spp. [35]. In the view of the fungi, the most common species detected in the lung microbial community include *Aspergillus*, *Penicillium*, *Cryptococcus*, *Eurotium*, and *Candida*, with *Candida* species predominating [36].

Lung microbiota exhibits significant compositional similarity to oral bacterial communities but with lower concentration, while differing considerably from nasal microbiota, supporting the concept of a continuous microbial axis extending from the mouth to the lungs and gut [37]. Bassis et al. [37] demonstrated that the observed lower airway microbiota was unlikely to result from upper airway contamination by using bronchoalveolar lavage (BAL) with contamination controls, including bronchoscope rinse and saline controls, and serial BAL sampling. However, these findings are based on microbial DNA detection, which is culture-independent sequencing rather than viable organism culture and therefore reflects bacterial presence rather than confirmed microbial viability [35,36,37].

The composition and diversity of the lung microbiota change dynamically through microbial immigration, replication, and elimination processes. Microbial immigration is the result of micro-aspiration of saliva, which contains a sufficient amount of bacteria, or dispersion from the oropharynx or nasopharynx into the respiratory tract [31]. Although unproven, physiological micro-aspiration is thought to occur predominantly during sleep, when supine posture and reduced laryngeal and cough reflex activity favor aspiration [38]. Active replication of the microbiota occurs despite various pulmonary environmental factors, including high surfactant levels, oxygen tension, redox status, and mucosal pH, etc. Elimination by coughing, mucociliary clearance, and immune defense are also important processes for maintaining the balance of microbial biomass and maintaining gas exchange in the lung. Also, these microbiotas usually transiently colonize rather than reside in healthy individuals. The number of microbes present in the lungs is determined by the previous three factors. However, in healthy lungs, the microbial composition is mostly determined by immigration and elimination [34]. If there is a dramatic increase in immigration or decrease in elimination, it may lead to dysregulation of airway immunity, progressive lung injury, and inflammation [31,34]. In contrast to chronic lung disease, the structure of the pulmonary microbiome becomes increasingly shaped by local environmental conditions and species-specific growth advantages rather than the immigration and elimination processes. Multiple changes, such as anoxia from excessive mucous production, increased vascular permeability, and increased inflammatory cells and their inflammatory by-products, including cytokines, catecholamines, and temperature of the airway and alveoli during lung diseases, might be selective for growth of some bacterial species, e.g., *Pseudomonas aeruginosa*, *Streptococcus pneumoniae*, *Staphylococcus aureus*, *Burkholderia cepacian* complex. In summary, persistent bacterial colonization is the bacterial species that have adapted to the altered, injured pulmonary environment [34].

In the long term, in normal homeostasis, microbial colonization delivers essential factors that drive the maturation of local immune cells involving both innate and adaptive immunity [13,39]. The absence of lung-specific microbial communities has been associated with increased Th17- and neutrophil-dominant mucosal immune profiles and reduced innate immune activity, indicating that the lung microbiota may exert important immunomodulatory effects [40].

## 4. Mechanistic Evidence for Bidirectional Gut–Lung and Lung–Gut Communication

### 4.1. Gut-Lung Axis

The first model of the gut-lung axis was introduced in 1990, describing the development of acute respiratory distress syndrome (ARDS) following septic shock. In this model, bacterial products translocate from the intestinal lumen into the bloodstream due to increased intestinal mucosal barrier permeability. The substances pass through the liver and activate Kupffer cells, leading to stimulation of inflammatory mediators such as TNF-α, IL-1, and IL-6 that trigger neutrophil degranulation in the lung, leading to ARDS [41].

**Figure 1:**
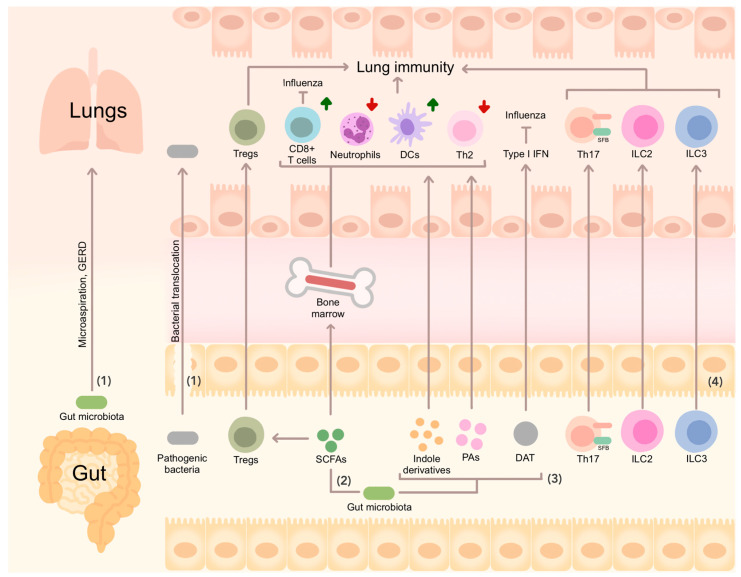
**The gut-lung axis.** Direct communication between the respiratory and gastrointestinal tracts occurs by micro-aspiration, GERD, and bacterial translocation from the intestinal tract into the circulation and targeting the lung (**1**). Unmetabolized SCFAs enter the circulation and bone marrow, resulting in the priming of immune cells, which leads to pulmonary infection defense and anti-inflammation (**2**). Other gut microbiota-derived substances, such as indole derivatives, PA, and DAT, also play important roles in anti-inflammatory response and lung protection (**3**). Multiple immune cell migrations, including Th17, ILC2, and ILC3 cells from the gut to the lung, affect pulmonary immunity in various ways (**4**). Brown solid arrows mean conceptual mechanisms. Short green arrows mean increase and short red arrows mean decrease. DAT, desaminotyrosine; GERD, gastroesophageal reflux; ILC, innate lymphoid cells; LPS, lipopolysaccharide; PA, polyamine; SCFA, short-chain fatty acid; Th17, T helper type 17.

Emerging evidence indicates that dysbiosis of microbial communities in both the gut and lungs is linked to the pathogenesis of acute and chronic pulmonary diseases [31,42]. This interaction is bidirectional; however, the gut–lung axis generally exerts a stronger influence than the lung–gut axis [42]. The lungs and gut share a common endodermal origin and are composed of columnar epithelial cells equipped with microvilli in the gut or cilia in the respiratory tract. These specialized cells serve as both mechanical and immune barriers, acting in concert with associated lymphoid tissues to preserve mucosal immune homeostasis. Both the gut and lungs secrete mucus via goblet cells and release secretory IgA; however, IgA production is less abundant in the pulmonary tract than in the intestine [2]. The gut-lung axis interaction is demonstrated in Figure 1.

#### 4.1.1. Direct Communication of Gut-Lung Axis

The oral cavity serves as a common anatomical gateway for both the gastrointestinal and respiratory tracts. Lung microbiota exhibits significant compositional similarity to oral bacterial communities but with lower concentration, while differing considerably from nasal microbiota, supporting the concept of a continuous microbial axis extending from the mouth to the lungs and gut [37,42]. This phenomenon can be explained by the migration of microorganisms from the oral cavity to the gastrointestinal tract through swallowing, followed by some micro-aspiration (Figure 1(1)) [37]. This event allows some bacteria to also be inhaled into the lungs. Other mechanisms, for example, in cases of gastroesophageal reflux (GERD), gastric contents can regurgitate into the mouth and subsequently be aspirated into the lungs (Figure 1(1)) [42]. Another mechanism mentioned is bacterial translocation from the intestinal lumen into the bloodstream during inflammation and intestinal barrier disruption (Figure 1(1)) [41].

#### 4.1.2. Indirect Communication and Immune Modulatory Pathway of the Gut-Lung Axis

Gut microbiota-derived metabolites, including SCFAs and amino acid derivatives, are absorbed by enterocytes and enter the portal circulation. After the first-pass metabolism by the liver, they enter the systemic circulation and distribute to all organs, including the lungs. Because of the large surface area of the blood-gas interface in the lungs, the lungs are highly effective at receiving circulating signals [24]. Another pathway of transferring immunomodulatory molecules, lipids, and immune cells is the mesenteric-thoracic duct pathway. The intestine is drained by the mesenteric system; unlike the portal vein, mesenteric lymph nodes drain into the cisterna chyli, subsequently into the thoracic duct, and finally mix with the left subclavian vein, bypassing the liver entirely. Lastly, the venous blood is pumped into the pulmonary circulation and enters the lungs [43]. In some cases, pathogenic intestinal bacteria can translocate across the gut barrier into the systemic circulation via the mesenteric pathway and subsequently trigger pulmonary inflammation upon reaching the lungs via this lymphatic pathway [44]. Furthermore, inflammatory gastrointestinal disease increases systemic inflammatory mediators that may disseminate to the lungs, thereby shaping immune response magnitude and plasticity [18].

There are various other pathways of gut-lung axis communication in the view of regulating immune cell migration between these sites [44]. Gut microbiota and their metabolites promote B- and T-cell expansion within Peyer’s patches and mesenteric lymph nodes and may be critical for directing immune cell trafficking from the intestine to the lungs [26]. Gut microbiota modulates immune function via local and systemic mechanisms that engage CD8^+^ T cells, Th17 cells, multiple ILs, and NF-κB –dependent signaling pathways [44]. A balanced gut microbial community enhances host resistance to respiratory bacterial and viral infections [42]. Additionally, dysbiosis of these microbiota leads to an inflammatory response resulting in lung injury, pulmonary fibrosis, and carcinogenesis [44].

##### Systemic Transfer of Microbial Substances

Direct effect of the gut microbiota on modulation of lung immunity: for example, the presence of microbial components is transported to the lungs via the circulation. Regarding experiments in animal models, intraperitoneal injection of LPS in antibiotic-treated mice revealed restoration of an effective immune response to influenza lung infection by increased expression of mRNA for pro–IL-1β and pro–IL-18 at steady state [45].

##### Gut Microbiota-Derived Metabolites

Short-chain fatty acids (SCFAs)

Not only microbial components that could cause an impact on pulmonary immunity transferred via the systemic circulation, but microbiota-related metabolites also have similar modulatory effects. Unmetabolized SCFAs that have not been used by the gastrointestinal tract are reported to be transported to the systemic circulation and are involved in the process of peripheral organs’ immune modulation, including bone marrow (Figure 1(2)) [18]. Regarding influenza pulmonary infection in an animal model, administration of diet-derived SCFAs, especially butyrate, modifies bone marrow hematopoiesis, leading to enhanced lymphocyte antigen 6 complex, locus C1 (Ly6c)- patrolling monocytes generation. This results in decreased C-X-C motif chemokine ligand (CXCL) 1 production in the airway, which eventually attenuates neutrophil recruitment [46]. Beyond localized effects, as mentioned, gut-derived Tregs, via inhibition of HDAC, transit through systemic circulation to the lungs, where they attenuate inflammation [24]. They also stimulate CD8+ T cell responses, simultaneously. This balances the effects of both innate and adaptive immunities, resulting in promotion of influenza infection resolution with less immune-mediated pathology [46]. Acetate has been shown to augment both the number and function of the Tregs via epigenetic effects, resulting in suppression of allergic airways disease [47]. Oral administration of propionate modulated bone marrow hematopoiesis through GPR41/FFAR3 on the macrophage and dendritic cell progenitors leading to increased numbers of these cells and enhanced pulmonary phagocytic activity while suppressing T helper type 2 (Th2) responses, resulting in preventing allergic inflammation and subsequent asthma [48] SCFAs also regulate the alveolar macrophages via GPR41 and GPR43 by enhancing the IL-10 secretion while inhibiting the pro-inflammatory cytokines including TNF-α, IL-1β, and IL-6 [24]. SCFAs treatment in mice showed fewer IL-4-producing CD4+ T cells and circulating immunoglobulin E (IgE), the underlying mechanism of allergic response and inflammation [49]. Moreover, in germ-free mice, more lymphocyte and eosinophil infiltration, Th2-associated cytokines, and IgE levels leading to allergic airway inflammation are documented [50]. In general, SCFAs strengthen the pulmonary epithelial barrier integrity through the phosphorylation of the tight-junction protein, including occludin and claudin [51].

Indole derivatives

Indole derivatives, once they enter the systemic circulation and reach the lungs, these substances act as ligands for the aryl hydrocarbon receptor (AhR), a ligand-activated transcription factor expressed by immune cells and epithelial cells. Upon ligand binding, AhR is translocated to the nucleus and drives the expression of immunosuppressive transcriptional processes by modulating functions of dendritic cells, macrophages, T cells, and B cells, resulting in an immunosuppressive state to attenuate lung inflammation (Figure 1(3)) [52].

Polyamines (PA)

PAs are substances converted from arginine by multiple gut microbiota, for example, *E. coli* [53]. Their functions are stimulation of the phagocytic activity of the alveolar macrophages; attenuation of inflammatory cytokines, including TNF-α, IL-1β, and IL-6; and antioxidation of pulmonary free radicals from smoking (Figure 1(3)) [24].

Desaminotyrosine (DAT)

DAT is a by-product of flavonoids produced by gut microbiota, especially *Clostridium orbiscindens*, which enhances type I interferon (IFN) signaling and results in influenza defense (Figure 1(3)) [54].

##### Segmented Filamentous Bacteria (SFB)

The presence of SFB in the gut, either through natural colonization or probiotic supplementation, has been shown to induce higher levels of IL-22, larger numbers of IL-22(+) TCRβ(+) cells, and Th17 cells via CD4+ T-cell polarization, with lower infection severity of *Staphylococcus aureus* pulmonary infection, bacterial burdens in the lungs, lung inflammation, and mortality [55]. Th17 cells also stimulate antimicrobial peptides such as β-defensin 2 to enhance mucosal immunity [44]. However, higher Th2 and Th17 cells were found in asthma [56]. Administration of herbal agents that modulate gut microbiota composition increases circulating propionate and butyrate levels, suppresses TLR4/MyD88/NF-κB signaling in both lungs and intestinal tissues, and inhibits Th2 and Th17 cell migration [57]. In autoimmune contexts, SFB drive robust Th17 differentiation, with systemic expansion and preferential lung homing mediated by pulmonary chemokines such as chemokine C-C motif ligand (CCL) 20 [58]. Pathogenic dual TCR–expressing Th17 cells capable of recognizing both SFB-derived peptides and self-antigens further amplify autoimmune inflammation, particularly in the lung [58].

##### Migration of Immune Cells

There is also evidence of direct migration of immune cells between organs, including the intestine and lungs (Figure 1(4)). ILCs are T cell lymphocytes found in the intestinal lamina propria involved in host defense, tissue repair, metabolic regulation, and inflammation [59]. During parasitic infection, IL-33 and IL-25 mediate ILC2 expansion, and proliferating ILC2 secrete IL-5 and IL-13 to support host resistance [26,60]. Lung ILC2 may originate from local pulmonary proliferation or from peripheral circulation, including populations derived from the intestine [61]. Intraperitoneal injection of IL-25 induced the circulation of ILC2 cells from the intestine to the lung tissue [59]. Lung-migrating ILC2 secrete cytokines essential for host defense and immune-mediated tissue damage control. However, ILC2 presentation was associated with many allergic disorders and eosinophilic inflammation, including allergic rhinitis, asthma, atopic dermatitis, and food allergies [60]. Butyrate, but not acetate or propionate, suppressed IL-5 and IL-13 production by murine ILC2s, and both systemic and local butyrate administration markedly reduced ILC2-driven airway hyperresponsiveness and inflammation [62]. IL-1β and IL-23–activated ILC3s produce IL-17 and IL-22, contributing to immunity against extracellular bacteria and fungi. Pulmonary ILC3 accumulation reflects cellular trafficking rather than local proliferation [26]. Detection of commensal bacterial colonization or the presence of butyrate is the stimulant for the migration of IL-22-producing ILC3 into the lungs, which has an essential role for pneumonia prevention [63].

### 4.2. Lung-Gut Axis

**Figure 2:**
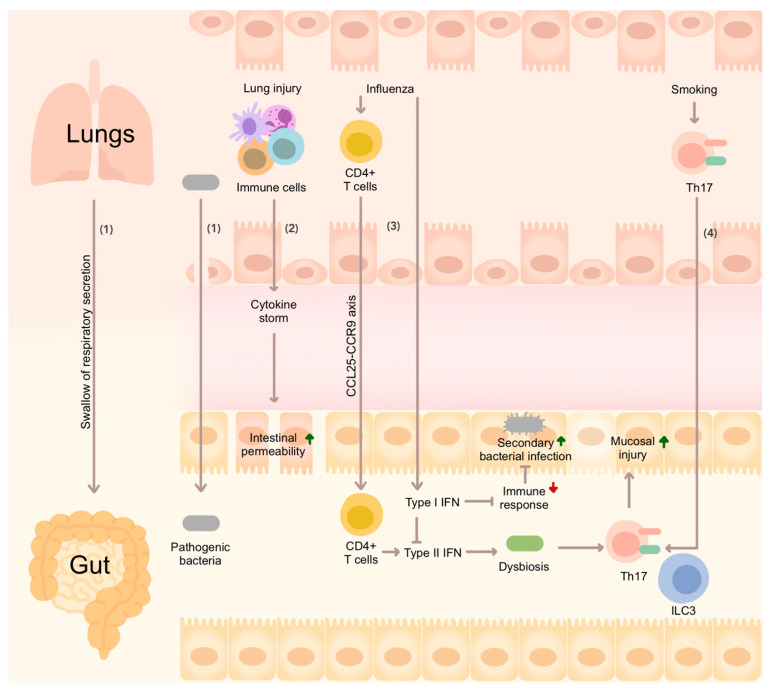
**The lung-gut axis.** The direct interaction between the respiratory and gastrointestinal tract occurs by swallowing respiratory secretions and translocation of pulmonary pathogens into the systemic circulation during lung insult, which eventually reaches the gastrointestinal tract (**1**). Severe lung injury triggers a massive release of pro-inflammatory cytokines into the bloodstream, resulting in gut injury (**2**). An experimental model of influenza infection leads to immune cell migration via the CCL25–CCR9 chemokine axis and involvement of IFN, leading to intestinal dysbiosis, local immune suppression, and intestinal injury (**3**). In experimental models, smoking is responsible for inflammation in the gut via migration of Th17 cells (**4**). Brown solid arrows mean conceptual mechanisms. Short green arrows mean increase and short red arrows mean decrease. CCL25-CCR9, chemokine C-C motif ligand 25- chemokine C-C motif receptor 9; IFN, interferon. Th17, T-helper.

The lung-gut axis demonstrates a bidirectional relationship where respiratory conditions might also result in changes in gut homeostasis and gut microbiota communities, which is also known as the lung-gut axis. Compared with gut-to-lung communication, the reverse lung–gut axis remains less clearly defined, with sparse information limited to lung injury and infection. The lung-gut axis acts through multiple mechanisms, including direct physical interaction, bacterial translocation, systemic inflammation, and immune cell trafficking. The main pathway of lung-gut axis communication is demonstrated in Figure 2.

#### 4.2.1. Direct Communication of the Lung-Gut Axis

Regarding this lung–gut axis, one proposed mechanism involves the most direct physical transmission of secretion of substances, pathogens, and inflammatory cytokines from the lungs that are cleared through mucociliary transport. This process moves mucus from the lower respiratory tract toward the pharynx and is subsequently swallowed or expectorated (Figure 2(1)) [42].

Another pathway involves the translocation of pulmonary bacteria into the systemic circulation following severe lung injury, with subsequent dissemination to the intestine. In an animal study, acute pulmonary LPS instillation rapidly altered lung microbiota communities and induced neutrophil recruitment. While lung bacterial communities were slightly nonsignificantly reduced from 4 to 48 h, followed by recovery at 72 h, increased bacterial loads with similar communities as the bronchoalveolar lavage were also detected in the blood. Furthermore, the cecum showed a significant rise in total bacterial load, but without changes in diversity. Together, these support the concept of bacterial translocation from the injured lung into the bloodstream and consequently reach distant organs, including the intestine (Figure 2(1)) [64].

#### 4.2.2. Indirect Communication and Distant Immune Modulatory Pathway of the Lung-Gut Axis

##### Cytokine Storm

Indirect mechanism occurs through severe lung inflammation, which triggers a systemic cytokine storm that damages the intestinal barrier. In conditions such as ARDS or other forms of lung injury, pulmonary capillary damage occurs, leading to the migration of immune cells into the lungs and the subsequent release of large amounts of pro-inflammatory cytokines, including TNF-α, IL-1β, IL-6, and IL-8, into the systemic circulation. When these cytokines reach the gut, they induce functional alterations in tight-junction proteins, such as claudins and occludin, and activate myosin light chain kinase, resulting in marked intestinal hyperpermeability. Furthermore, pro-inflammatory cytokines, including TNF-α, can bind to TNF receptors on intestinal epithelial cells and promote local inflammation through the NF-κB pathway, further exacerbating intestinal barrier disruption (Figure 2(2)) [65].

##### Infection- and Inflammation-Induced Immune Cells Migration and Gut Microbiota Modulation

Another indirect mechanism linking lung injury to intestinal inflammation involves lung-derived immune cell migration, gut microbiota alteration, and subsequent immune activation. In an animal model of influenza lung infection, intestinal injury was not caused by direct viral infection of the gut [66,67]. Instead, lung injury activated CCR9+CD4+ T cells in the lungs, which then migrated to the intestine through the chemokine C-C motif ligand 25- chemokine C-C motif receptor 9 (CCL25–CCR9) axis. CCL25 is highly expressed by intestinal epithelial cells, but not by non-mucosal organs such as the liver and kidney, and specifically guides CCR9-expressing effector lymphocytes to the small intestine (Figure 2(3)) [66]. Once in the intestine, these lung-derived CD4+ T cells secrete IFN-γ, a type II IFN, thereby disrupting microbial homeostasis [66]. This dysbiosis is characterized by reduced SFB and *Lactobacillus* abundance, along with increased *Enterobacteriaceae* including *E. coli* [66,67]. SFB are *Clostridia*-related bacteria that closely adhere to the intestinal epithelium and support host defense through antimicrobial production and enhanced colonization resistance against intestinal pathogens [67]. *E. coli*, in contrast, stimulates IL-15 expression in intestinal epithelial cells, which promotes Th17 polarization and contributes to intestinal immune injury [66]. The enrichment of these *Proteobacteria* has been observed in various human intestinal inflammatory disorders such as Crohn’s disease and enteropathy in individuals infected with human immunodeficiency virus (HIV). Influenza-induced type I IFN also contributes to this process by promoting gut dysbiosis and increasing susceptibility to secondary enteric bacterial infection, particularly by *Enterobacteriaceae*. This occurs through suppression of protective intestinal immune responses, including reduced IFN-γ antibacterial activity, decreased IL-6 and CXCL2 levels, impaired macrophage activation and neutrophil recruitment, and increased IL-10, ultimately weakening intestinal immunity and promoting bacterial dissemination (Figure 2(3)) [67].

Smoking has also been shown to have a similar pathway of inflammatory response through increased Th17 cells in not only the lungs, but also in the circulation and intestine. These cells produce IL-17A, which promotes neutrophil recruitment and local inflammation. This response is accompanied by increased IL-1β and reduced anti-inflammatory IL-10 levels. In the gut, increased ILC3s also produce IL-17A, further amplifying intestinal inflammation, immune cell infiltration, and histopathological colitis scores (Figure 2(4)) [68].

Clinical studies further support the presence of gut dysbiosis during respiratory viral infections. Using 16S rDNA sequencing of fecal samples, altered intestinal bacterial richness and diversity have been observed in patients with COVID-19 and influenza A/H1N1 pulmonary infection compared with healthy controls [69]. Patients with COVID-19 showed reduced intestinal bacterial diversity, increased relative abundance of opportunistic pathogens such as *Streptococcus*, *Rothia*, *Veillonella*, *Actinomyces*, *Clostridium*, and *Bacteroides*, and decreased abundance of beneficial symbionts [69,70]. In contrast, patients with H1N1 infection showed reduced abundance of anaerobic butyrate-producing bacteria, including *Lachnospiraceae* and *Ruminococcaceae*, along with increased opportunistic pathogens such as *Enterococcus*, *Prevotella*, *Finegoldia*, and *Peptoniphilus*. The relative abundance of *Streptococcus* and *Escherichia/Shigella* is also significantly higher in COVID-19 and H1N1 patients, respectively [69].

A further consequence of gut dysbiosis caused by viral respiratory infections is a significant reduction in SCFAs, the well-known energy source for intestinal cells. This metabolic disturbance may affect the production of alpha-galactosylceramides, lipid ligands recognized by invariant natural killer T cells, which then potentially impairs these cells’ immune regulation [71]. Moreover, changes in intestinal oxygen availability, together with systemic hypoxia caused by respiratory failure, may shift the gut environment toward the expansion of facultative anaerobes and opportunistic pathogens [65,71].

## 5. Evidence Synthesis of Plant-Derived Modulation of the Gut–Lung Axis

### 5.1. Plant-Based Substances

A basic plant-based diet is characterized by intake of complex, largely indigestible plant-derived substances, particularly dietary fibers, polysaccharides, and polyphenols. Dietary fibers comprise polymers of monosaccharides that are not digested or absorbed in the gastrointestinal tract because humans lack the necessary hydrolytic enzymes. Dietary fibers consist of resistant oligosaccharides, resistant starches, and non-starch polysaccharides, as well as fiber-associated non-carbohydrate components such as lignin, cutin, and suberin. These components can be found in grains, fruits, and vegetables and also in fungi and algae [5]. Resistant or low-molecular-weight non-digestible oligosaccharides include fructooligosaccharides (FOS), galactooligosaccharides (GOS), and xylooligosaccharides (XOS). Resistant starches include physically inaccessible starch, native granular starch, retrograded starch, chemically modified starch, and amylose–lipid complexes. Non-starch polysaccharides include cellulose, hemicellulose, pectins, beta-glucans, gums, mucilages, galactomannans, and inulin [5,72]. Polyphenols are plant secondary metabolites that are classified into flavonoids, such as flavones, flavonols, isoflavones, flavanones, flavanols, and anthocyanidins, as well as non-flavonoids, such as lignans, phenolic acids, stilbenes, and tannins [73]. Polyphenols can be found in fruits, vegetables, cereals, beans, tea, coffee, honey, red wine, olive oil, and whole grains [74]. Simultaneously, gut bacteria also metabolize plant components and dietary amino acids into other secondary metabolites, including tryptophan derivatives and modified bile acids, which eventually interact with host receptors to maintain immune homeostasis [75]. Collectively, these compounds represent key nutritional substrates in plant-based diets and may contribute to host health through their interactions with the gut microbiota and systemic involvement, as these microbial metabolites do not remain confined to the intestinal tract. These metabolites can disseminate systemically through the bloodstream to influence distal organs, including the lungs [76]. This forms the basis of the gut-lung axis, a bidirectional communication network where gut-derived metabolites dictate the immunological environment of the respiratory tract and regulate pulmonary immune responses against environmental antigens.

### 5.2. Mechanistic Evidence for Plant-Based Substances

The prebiotic properties of dietary fiber arise from its ability to reach the large intestine undigested, where it serves as a substrate for beneficial gut bacteria. Through fermentation, these bacteria increase in both growth and activity [77]. Notably, plant-derived polysaccharides and polyphenols are associated with a wide range of biological activities, such as antioxidant, anti-inflammatory, immune-regulating, antitumor, and gut microbiota–modulating effects [5]. Various studies regarding plant-based substances have demonstrated their roles in respiratory diseases potentially via the gut-lung axis through several mechanisms, as summarized in Table 1.

#### 5.2.1. Microbiota-Dependent Mechanisms

##### SCFAs Production

One of the most plausible mechanisms linking plant-derived substances to gut–lung communication involves the production of SCFAs through microbial fermentation of dietary fibers and other non-digestible carbohydrates [113]. These SCFAs are crucial signaling molecules in host–microbe interactions and may contribute to gut-to-lung immune regulation as mentioned in the above section. The SCFAs’ actions include inhibition of pro-inflammatory cascades, attenuation of inflammatory cytokine production, and promotion of anti-inflammatory responses, including stimulation of Treg differentiation and function and anti-inflammatory cytokines such as IL-10 via various GPR interactions, thereby suppressing airway inflammation [114]. In experimental models, SCFAs have been associated with reduced airway inflammation and lung injury. For example, SCFA-related signaling has been reported to attenuate LPS-induced acute respiratory distress syndrome through inhibition of the Janus kinase 2/signal transducer and activator of transcription 3 (JAK2/STAT3) pathway [108]. In allergic airway inflammation models, fermentable fibers or SCFAs were associated with reduced airway hyperresponsiveness and lower Th2-related markers, including IgE, IL-4, IL-5, and IL-13 [82,88,90]. Microbial butyrate has also been shown to suppress ILC2 function by downregulating GATA-3 transcription, thereby reducing ILC2-dependent airway hyperreactivity and eosinophilic inflammation in experimental asthma models [72]. These findings support SCFAs, metabolites from many plant compounds, as important candidate mediators of gut–lung interaction, although the degree of direct causality varies across studies and needs further validation.

However, high-cellulose intake improved airway inflammation in asthmatic mice without increasing intestinal SCFA levels, leading the authors to propose that insoluble fiber may modulate pulmonary inflammation through lipid-metabolic pathways that influence Th2-cell differentiation and inflammatory responses [82]. Other gut microbiota-related pathways rather than SCFAs are also considered.

##### Microbiota Modulation

A plant-based diet can induce rapid changes in gut microbial communities, sometimes within 24 h after dietary transition [115]. In respiratory disease models, high-fiber diets, polysaccharides such as pectin, and polyphenol-containing interventions have been reported to decrease the *Firmicutes*/*Bacteroidetes* balance [47,48,84,91,94,95,102,105]. *Bacteroidetes* is known to be a potent SCFAs fermenter and has been considered a beneficial gut microbiota, while *Firmicutes* has been revealed in previous studies to be abundant in Western high-fat diet consumers, chronic obstructive pulmonary disease (COPD) patients, and other intestinal inflammation diseases [47,94,102]. Therefore, this finding may suggest the benefits of the plant-based intervention in modulating gut microbiota communities.

Plant-derived substances may also alter gut microbial composition by reversing disease-associated gut dysbiosis and selectively enriching beneficial bacterial taxa, including other SCFA-producing and inflammation-suppressing microbes [48].

For example, polyphenols such as resveratrol, epigallocatechin-3-gallate (EGCG), and baicalein have been associated with increased *Akkermansia muciniphila,* a gut microbiota capable for producing SCFAs, in both the intestine and lungs, resulting in higher propionate or butyrate levels in experimental models, along with improved epithelial barrier markers and reduced allergic or acute lung inflammation [75,88,91,103,108]. Baicalein, a flavone derived from *Scutellaria baicalensis*, was reported to enrich *Prevotellaceae*, another gut microbiota with SCFA-producing capability, which modulates FFAR2/FFAR3 and TLR4/MyD88-related signaling in acute lung injury models [111].

Polysaccharides have been reported to reduce potentially pathogenic *Proteobacteria*, a phylum often associated with intestinal dysbiosis and opportunistic pathogens [84,102,105]. *Ephedra sinica* polysaccharides were associated with increased *Bacteroides*, *Lactobacillus*, and *Prevotella*, higher SCFA availability, reduced NF-κB signaling, and attenuation of PM2.5-aggravated asthma-like inflammation [80]. *Houttuynia cordata* polysaccharides (HCP) were reported to reshape gut microbiota and modulate the Th17/Treg immune balance, which begins first in GALT and finally in the lungs, in H1N1-induced viral pneumonia models. Furthermore, depletion of gut microbiota by antibiotic cocktails abolished the protective effects of HCP against intestinal and lung injury [98]. This finding suggests that the therapeutic effects of HCP may partly depend on the presence of gut microbiota. Additionally, no change was observed in non-mucosal organs including liver, kidney, and spleen, supporting the gut–lung axis of mucosal organs interaction theory [99]. Moreover, experimental models of maternal supplementation with inulin, which is enriched in multiple fruits and vegetables, have been shown to increase SCFA-producing gut microbiota and circulating SCFAs; these microbial metabolites may traverse the placental barrier and directly shape offspring immune development. This transplacental influence may contribute to improved Th1/Th2 balance through SCFA receptor signaling, particularly GPR41, thereby attenuating allergic airway inflammation in offspring [85]. Therefore, various gut microbiota-related metabolic pathways may be involved in the change in inflammatory responses, rather than SCFAs alone. Overall, these studies suggest possible microbiota-linked mechanisms.

#### 5.2.2. Microbiota-Independent Mechanisms

Some plant-derived substances may influence respiratory inflammation through mechanisms that are partly independent of gut microbiota composition or SCFA production [78,79]. In ovalbumin-sensitized models, indigestible oligosaccharides such as raffinose and α-linked galactooligosaccharides reduced allergic airway eosinophilia and pulmonary IL-4 and IL-5 expression, suggesting attenuation of Th2-driven inflammation. Importantly, these effects persisted after cecectomy and neomycin treatment, which markedly reduced colonic bacteria, and were also observed after intraperitoneal administration [78,79]. These findings support a possible microbiota-independent or post-absorptive immunomodulatory mechanism [79]. Consistent with this interpretation, raffinose was detected in portal and systemic circulation after oral administration, suggesting that absorbed oligosaccharides may directly influence immune-cell trafficking or cytokine expression in the inflamed lung [78].

### 5.3. Plant-Based Substances as Therapeutic Strategies

The translational relevance of plant-derived modulation of the gut–lung axis is promising but remains incompletely established. Epidemiological and experimental evidence suggests that plant-rich dietary patterns, such as the Mediterranean diet, which provides fibers, polysaccharides, and polyphenols, are inversely associated with asthma and other allergic diseases [116]. Although plant-derived fibers, polysaccharides, polyphenols, and flavonoids are promising modulators of gut microbiota, microbial metabolites, and inflammatory pathways, their use as therapeutic strategies requires cautious interpretation. Most available evidence is derived from animal models using specific doses, routes of administration, and preparation that may not reflect usual human dietary intake or clinical phytotherapy.

#### 5.3.1. Dose Translation

Human dose standardization is generally lacking in most studies, except for some plant-derived compounds that have already been used in humans. For example, in the study of partially hydrolyzed guar gum (PHGG), the authors stated that the 500 mg/kg mouse dose was estimated to correspond to approximately 2000 mg/day in humans after accounting for mouse–human metabolic differences, and this was supported by previous human use of PHGG at 5–10 g/day for diarrhea or constipation [96]. In other studies, the mouse-equivalent dose was calculated from the human dose using body surface area conversion [87,107]. However, these dose conversions do not necessarily confirm that similar biological or respiratory effects would occur in humans, and many further compounds still need dosage validation. Therefore, further clinical studies are needed to determine clinically relevant dosing, efficacy, and safety.

#### 5.3.2. Safety Concerns

Safety reporting regarding these plant-based substances was limited in most studies. For EGCG, one study reported dose-related safety or acute toxicity data in mice, suggesting tolerability at doses ≤ 20 mg/kg; however, corresponding human dosage or safety information was not provided [108]. In contrast, the inulin study reported mild gastrointestinal symptoms, including indigestion with inulin and diarrhea with inulin plus probiotics, but no participants withdrew because of gastrointestinal symptoms or other adverse events [86]. Overall, available safety data remains incomplete, particularly regarding clinically relevant dosing and tolerability in humans.

## 6. Discussion

The evidence summarized in this review supports the gut–lung axis as a biologically plausible communication network between intestinal microbiota, microbial metabolites, mucosal immunity, and pulmonary inflammation. Among the pathways discussed, gut microbiota and their metabolites, particularly SCFAs, play a critical role in the gut- lung axis [25]. Dietary fibers, resistant starches, oligosaccharides, and polysaccharides can be fermented by gut microbiota to generate SCFAs, particularly acetate, propionate, and butyrate. SCFA-mediated immune modulation through GPR (FFAR) and HDAC inhibition is one of the most consistently supported mechanisms [117].

In contrast, the lung–gut axis remains less clearly established. Evidence that respiratory infection, lung injury, or smoking can alter gut microbiota composition and intestinal barrier function is biologically plausible and supported mainly by preclinical models of acute injury or infection, with only a limited number of supporting clinical studies. Therefore, lung–gut communication should be interpreted as an emerging component of the bidirectional mucosal network.

Plant-derived substances have attracted increasing attention in gut–lung axis research because they can alter gut microbial composition and may serve as substrates for microbial SCFA production. However, many studies reported increased SCFA-producing bacteria, shifts in the *Firmicutes*/*Bacteroidetes* ratio, or enrichment of beneficial taxa such as *Bifidobacterium*, *Lactobacillus*, *Akkermansia*, or *Bacteroides* alongside improved lung inflammation, rather than providing a direct causal relationship [81]. Regarding the *Firmicutes*/*Bacteroidetes* ratio, although a higher *Bacteroidetes*/*Firmicutes* ratio has been negatively associated with dysbiosis in some settings [48], increased *Firmicutes* abundance after plant-derived interventions should not automatically be considered unfavorable [89,106]. In some disease models, *Firmicutes* may be reduced as part of disease-associated dysbiosis, and their restoration may reflect recovery of microbial balance rather than worsening inflammation [89,106]. Moreover, several *Firmicutes* taxa are important butyrate producers, suggesting that some increases in *Firmicutes* may contribute to enhanced SCFA synthesis and anti-inflammatory effects [74]. Therefore, changes in the *Firmicutes*/*Bacteroidetes* ratio or other changes in microbiota should be interpreted in a context-dependent manner, considering the disease model, specific bacterial taxa involved, and associated functional outcomes rather than viewed as uniformly beneficial or harmful.

The mechanisms by which plant-derived substances induce these microbial changes also remain incompletely understood [108]. Although several studies have reported increased SCFA levels together with improvements in respiratory-related outcomes, others have shown nonsignificant SCFA changes. These discrepancies may reflect differences in sampling time, such as measuring plasma SCFAs after a 12 h fast and potentially missing the postprandial SCFA peak [86], as well as differences in fiber fermentability, since not all dietary fibers are equally fermented. Different dietary fiber types, depending on their fermentability, can produce distinct changes in metabolite profiles and influence the levels of specific SCFAs. For example, pectin is generally more fermentable than cellulose and has been shown to increase SCFA production and tend to improve inflammatory markers more effectively [75]. In contrast, cellulose is an insoluble and less fermentable fiber that may shift the gut microbiota toward taxa that do not necessarily produce higher amounts of SCFAs [82]. In addition, plant-derived compounds may act through microbiota-independent mechanisms, including direct antioxidant effects, epithelial barrier regulation, inhibition of inflammatory signaling pathways, or post-absorptive immunomodulatory effects, as suggested by studies showing persistent respiratory effects even in microbiota-depleted models. Therefore, microbiota alterations alone do not establish that microbiota modulation is responsible for the observed pulmonary effects. Stronger evidence for a microbiota-mediated gut–lung mechanism would require additional approaches, such as antibiotic depletion, fecal microbiota transplantation, germ-free or microbiota-depleted models, metabolite supplementation, or specific receptor-knockout models. Taken together, current findings suggest that plant-derived substances may influence respiratory disease through multiple pathways, involving both microbiota-dependent and microbiota-independent mechanisms.

Based on the available evidence, it is difficult to determine which plant-derived substances have the greatest respiratory benefit. Overall, different plant-derived compounds appear promising in preclinical respiratory disease models, but these findings should be interpreted cautiously. Direct comparisons between different types of plant-derived interventions are still limited, and further studies are needed once their potential effects have been more clearly established. At present, the evidence remains insufficient to define a superior compound or resolve existing uncertainties.

Although human studies on plant-derived substances are increasing, clinical evidence specifically linking these interventions to gut–lung axis mechanisms remains limited. Most studies discussed in this review were conducted in animal models of ovalbumin- or house dust mite-induced allergic airway inflammation, cigarette smoke-induced COPD-like disease, acute lung injury, or viral infection. Although these models are valuable for mechanistic exploration, they do not fully capture the heterogeneity of human respiratory diseases. This limitation is partly due to the acute nature of many experimental models and the relatively short duration of treatment interventions [86,104,110]. In contrast, human respiratory diseases are usually chronic and also influenced by multiple factors including age, diet, medication exposure, environmental conditions, comorbidities, baseline microbiota composition, and disease phenotypes. In addition, many preclinical studies use prophylactic pretreatment designs, which may be difficult to translate into future clinical trials and are often less practical in real-world settings [91,108]. Therefore, beneficial effects observed in controlled animal models may not occur with the same magnitude, consistency, or direction in humans.

Dose translation is another important limitation. Many experimental studies use high doses of purified fibers, polysaccharides, polyphenols, or plant extracts that may not be achievable through ordinary dietary intake. Human dosage standardization also needs further studies for confirmation.

Another point of concern is the bioavailability of these plants. Despite low oral bioavailability of many polyphenols, particularly flavonoids, oral administration remains the practical route for administration; recent studies have focused on evaluating their effectiveness through this route [118]. Previous studies have reported favorable lung outcomes in respiratory disease models even through the oral route [111]. One possible explanation is that, after oral intake, flavonoids interact with the gut microbiota, where they may be fermented, degraded, and converted into more absorbable metabolites. This is supported by findings showing that flavonoid-derived metabolites can be detected in the circulation, whereas the parent compound remains largely confined to the intestine and is not detected systemically [119]. These microbial transformations may contribute to beneficial systemic effects and are often accompanied by changes in gut microbial composition and function, including alterations in SCFA production [111]. However, the exact bioavailability in humans still needs further studies.

Plant-derived foods and medicines are often perceived as safe. However, the composition of plant-derived preparations may vary according to plant species, extraction method, processing, storage, and quality control. Potentials of plant-based substance-drug interactions are also clinically relevant, especially in patients with asthma, COPD, infection, or acute lung injury who may receive inhaled corticosteroids, bronchodilators, antibiotics, anticoagulants, immunosuppressive agents, or other medications.

Therefore, dose equivalence, bioavailability, formulation, safety, and potential interactions with conventional respiratory medications should be considered before these interventions can be translated into clinical practice.

## 7. Conclusions

The gut–lung and lung–gut axes represent two directions of a bidirectional mucosal communication network linking microbial communities, microbial metabolites, epithelial barrier function, and immune-cell trafficking between the gastrointestinal and respiratory systems. Current evidence most consistently supports gut-to-lung mechanisms mediated by gut microbiota-derived metabolites, particularly SCFAs, which may regulate pulmonary immunity, host defense, and airway inflammation. In contrast, lung-to-gut mechanisms, including the effects of respiratory infection and lung injury on intestinal barrier function and gut dysbiosis, remain less clearly characterized and are supported mainly by preclinical and limited clinical evidence.

Plant-derived fibers, polysaccharides, and polyphenols represent promising modulators of microbiota-derived metabolites, epithelial barrier integrity, and immune-inflammatory responses. Their effects may be microbiota-dependent, such as through SCFA production and enrichment of metabolite-producing taxa, or microbiota-independent, such as through antioxidant activity, epithelial protection, and direct regulation of inflammatory signaling pathways. However, their use as established therapeutic strategies for respiratory diseases remains controversial, as clinical efficacy, optimal dose, safety in humans, bioavailability, and reproducibility remain insufficiently defined.

Future research should prioritize longitudinal human studies, standardized plant-derived preparations, integrated microbiome and metabolomic profiling, dose–response evaluation, biomarker validation, and controlled clinical trials in defined respiratory diseases, including asthma, COPD, viral respiratory infections, and acute lung injury. These approaches are needed to determine whether mechanisms identified in experimental models can be translated into safe, reproducible, and clinically meaningful interventions.

## Figures and Tables

**Table 1:** Effects of plant-derived substances on respiratory inflammatory diseases, potentially involving gut–lung-axis mechanisms.

Plant Sources	Respiratory Disease Model	Intestinal Microbiota Modulation	Lung Microbiota Modulation	Other Outcomes	Main Mechanism	Reference
Asthma						
Dietary fibers						
Raffinose (RAF) (oligosaccharide) 50 g/kg diet orally × 13 days or 0.5% (wt:v) solution (0.1 mL) i.p. × 13 days or 40% (wt:v) solution (4 g/kg BW) by gavage	OVA-induced allergic asthma (Brown Norway rats)	↑ total anaerobic bacteria (*Bifidobacteria*)	NR	↓ total cells and eosinophils in BALF both oral and i.p.; ↓ IL-4 and IL-5 mRNA in lung tissue; ↓ IgE; ↓ peribronchovascular inflammation in oral route Detected in portal and arterial plasma, peaking around 60 min after oral gavage	Microbial independent; post-absorptive mechanism	Watanabe et al. 2004 [78]
Raffinose (RAF)/GOS 50 g/kg diet orally or 0.5% (wt:v) solution (0.1 mL) i.p. × 13 days	OVA-induced allergic asthma (Brown Norway rats)	↑ total anaerobic bacteria (*Bifidobacteria*)	NR	↓ total cells and eosinophils in BALF both oral and i.p.; ↓ IL-4 and IL-5 mRNA in lung tissue in cecectomy + neomycin	Microbial independent	Sonoyama et al. 2005 [79]
*Ephedra sinica* polysaccharide (ESP) low, medium, high dose: 50, 100, 200 mg/kg, respectively by gavage × 12 days	PM2.5 and OVA-induced allergic asthma (BALB/c mice)	↑ *Bacteroides*, *Lactobacillus*, *Prevotella*, *Butyricicoccus*, *Paraprevotella*; ↓ *Enterococcus and Ruminococcus*	NR	↑ acetic, propionic, butyric, isobutyric, valeric, isovaleric, isohexanic acid; ↓ eosinophils in BALF; ↓ Ig-E, IL-6, TNF-α, IL-1β; ↓ lung tissue NF-κB; improve lung tissue damage and inflammation (dose-dependent)	NF-κB pathway regulation with microbial-related association	Liu et al. 2022 [80]
*Lycium barbarum* polysaccharide (LBP) 100 μg/g orally × 28 days	OVA-induced allergic asthma (C57BL/6 mice)	↑ *Lactobacillus*, *Bifidobacterium*; ↓ *Firmicutes*, *Actinobacteria*, *Alistipes*, *Clostridiales*	NR	↓ TNF, IL-4, IL-6, MCP-1, IL-17A in serum and BALF; ↓ lung injury in histopathology	Alleviating airway inflammation with microbial-related association	Cui et al. 2020 [81]
30% Cellulose (high fiber diet) orally × 28 days	OVA- and Al(OH)3-induced allergic asthma (C57BL/6J mice)	*Peptostreptococcaceae (family); Romboutsla, [Ruminococcus]_torques_group(genus)*	NR	↓ SCFAs; ↓ IgE, IL-4; improve airway epithelial structure, ↓ inflammatory cell infiltration around bronchus and blood vessel	Supportive microbial-related; lipid metabolism	Wen et al. 2022 [82]
Cellulose/Pectin (high fiber diet (30%)) orally × 3 days	Ozone-induced AHR (C57BL/6 mice)	↑ *Proteobacteria*, *Verrucomicrobia*; ↓ *Firmicutes* (Pectin) ↑ *Proteus*; ↓ *Lactobacillus*, *Parabacteroides*, *Clostridiales*, *Lachnospiraceae* (Pectin vs Cellulose)	NR	↓ ozone-induced AHR (cellulose) (male not female); addition of propionate did not alter O3-induced AHR	Sex-specific with microbial-related association	Tashiro et al. 2020 [83]
Cellulose/Pectin (4% fiber diet) + cellulose/pectin 0.4% of daily BW by gavage × 49 days	OVA-induced allergic asthma (BALB/c mice)	↑ proportion of *Bacteroidetes* and *Actinobacteria*; ↑ probiotic bacteria (*Lactobacillus* and *Bifidobacterium*); ↓ *Firmicutes* and *Proteobacteria*	NR	↓ allergic symptoms of nasal rubbing and sneezing; ↓ IgE; ↓ Th2 (IL-4) but ↑ Th1 cytokines (IFN-γ and IL-10) in NALF and BALF; ↓ eosinophil infiltration and goblet cell metaplasia in the nasal mucosa and lung tissue (pectin more effective)	Alleviating airway inflammation with microbial-related association	Zhang et al. 2016 [84]
Cellulose/Pectin (high fiber diet (30%)) orally × 3 weeks	HDM-induced allergic airway inflammation (C57BL/6 mice)	↑ proportion of *Bacteroidaceae* and *Bifidobacteriaceae;* ↑ *Bacteroidetes*/*Firmicutes* ratio	↑ *Bacteroidetes*/*Firmicutes* ratio	↑ SCFAs; ↓ total cells and eosinophils in BALF; ↓ IL-4, IL-5 (N.S.), IL-13 (N.S.), IL-17A in lung tissue; ↓ IgE; ↓ AHR	Direct microbial-related; SCFAs/GPR41 confirm with propionate supplement and alleviate response in GPR41-knockout mice	Trompette et al. 2014 [48]
High-amylose maize resistant starch orally × 16 days	HDM-induced allergic airway inflammation (C57BL/6 and BALB/C mice)	↑ *Bacteroidetes*; ↓ *Firmicutes*	NR	↓ total cells, eosinophil in BALF; ↓ IL-4, IL-5, IL-13, IL-10, IFN-γ; ↓ AHR	Supportive microbial-related; SCFAs	Thorburn et al. 2015 [47]
Recall high dietary fiber intake	Pregnant women without asthma (Observational study; N = 61)	NR	NR	Higher dietary fiber intake correlated with higher serum acetate only		
	Pregnant women without asthma (Observational study; N = 40)	NR	NR	Maternal acetate more than median associated with fewer infants needing ≥2 GP visits for cough/wheeze		
10% Inulin with drinking water orally × 1 week	OVA- and Al(OH)3-induced allergic asthma (Sprague-Dawley rats)	Maternal: ↑ SCFA-producing genera: mainly *Bifidobacterium* Offspring: *Enterobacteriaceae* dominant	NR	Maternal: ↑ SCFAs (acetic, propionic, isohexanoic acid) (N.S.) Offspring: ↓ IgE, IL-4, IL-17; ↑ IFN-γ, lung GPR41 mRNA	Supportive microbial-related; SCFA	Yuan et al. 2023 [85]
Inulin 12 g orally (twice daily) × 1 week	Stable asthma (3-way crossover trial; N = 17)	No difference in overall microbiota composition; ↑ relative *Bifidobacterium* (N.S.) (significant increase in inulin + probiotic group)	NR	No different change in SCFAs; improve ACQ6; ↓ sputum %eosinophils and HDAC9 gene expression; trend increase FEV1 (N.S.); FEV1 positive correlated changes in total fecal SCFA	Supportive microbial-related; HDAC-related anti-inflammation	McLoughlin et al. 2019 [86]
Polyphenols						
*Pyrus pyrifolia* cv. (pear extract) (ethanol extract) (Polyphenol)	OVA-induced allergic asthma (BALB/c mice)	NR	NR	↓ IgE, IL-4, IL-5, IL-13 (dose-dependent)	Supportive microbial-related	Yang et al. 2023 [87]
1 or 2 mg/kg by gavage × 8 days						
3 mL orally × 4 weeks	Mild asthma (RCT N = 20)	↑ *Bifidobacterium*, *Eubacterium*	NR	↓ urinary 2-NT and 1-OHP (positively associated with serum IL-4 and IgE, respectively)		
Baicalein (BAI) (Flavonoid) 10, 20, or 40 mg/kg by gavage × 15 days	OVA-induced allergic asthma (BALB/c mice)	↑ *Akkermansia muciniphila*	↓ *Alistipes* (Asthma associated)	↑ SCFAs (propionic acid); ↑ GPR41 mRNA and ↓ ILC2, TSLPR in colon tissue; ↓ eosinophil infiltration in lung tissue; ↑ TJ protein in lung and colon tissue; ↓ AHR (dose-dependent)	Direct microbial-related; SCFAs confirm with FMT after broad-spectrum antibiotics-induced microbiota depletion	Lu et al. 2026 [88]
*Spenceria ramalana* (ethanol extract) (Polyphenol) low and high dose: 200 and 500 mg/kg, respectively by gavage × 12 days	OVA + cigarette smoke-induced mixed asthma Sprague-Dawley rats	↑ *Firmicutes*; ↓ *Bacteroidetes*	NR	↓ total cells, eosinophils, neutrophils in BALF; ↓ IL-4, IL-5, IL-13, TNF-α; ↑ occludin, claudin-1, ZO-1 (N.S.) in the lungs; ↓ inflammation, collagen deposition, and mucus secretion in lung histopathology; ↓ p-Akt/Akt; ↓ wet/dry ratio of lung (dose-dependent)	PI3K/Akt signaling with microbial-related association	Xia et al. 2024 [89]
Ginger-processed Magnoliae Officinalis Cortex extract (GMOC) (ethanol extract) (Polyphenol) 1.3, 2.6, or 5.2 g raw herb/kg orally × 11 days	OVA-induced allergic asthma (C57BL/6 mice)	↑ *Lactobacillus*; ↓ *Allobaculum* and *Faecalibaculum*	NR	↑ SCFAs (acetic, propanoic, butanoic, and hexanoic acid); ↓ IL-4, IL-5, IL-13, IgE; ↓ inflammatory cell infiltration around airways and blood vessels; ↑ claudin-5, occludin, and E-cadherin in lung and colon; ↓ TRPA1, TRPM8, and TRPV4 (PI3K/AKT pathway) (dose-dependent)	Supportive microbial-related; SCFAs	Li et al. 2025 [90]
Honokiol 20 µM × 24 h	in vitro HEK293-TRPV1 cells	NR	NR	↓ TRPV1 (PI3K/AKT pathway)		
Resveratrol (RES) (Polyphenol) pretreatment 100 mg/kg by gavage x 14 days	OVA-induced allergic asthma (BALB/c mice)	↑ *Bacteroidetes* (*Bacteroidales*, *Bacteroides*); ↓ *Firmicutes*	↑ *Akkermansia muciniphila*	improve F, MV, sRaw, and dT; ↓ LPS in BALF; ↓ IL-13, IL-1β, IL-4, TNF-α, ↑ IL-10 in BALF and serum; ↑ occludin, claudin, E-cadherin, ZO-1 and ↓ mucin (Muc5ac) in lung	Supportive microbial-related; SCFAs, intestinal barrier function restoration	Alharris et al. 2022 [91]
50 µM × 48 h	in vitro murine lung epithelial cells	NR	NR	↑ occludin, claudin, E-cadherin, ZO-1, ZO-2, ZO-3		
Chronic obstructive pulmonary disease						
Dietary fibers						
*Ficus pumila L.* polysaccharides pretreatment Fruit polysaccharide (FFP); fruit aqueous extract (FPE); leaf polysaccharide (FPLP); leaf aqueous extract (FPLE) 50 mg/kg by gavage × 4 weeks	Cigarette smoke-induced COPD (ICR mice)	↑ SCFA-producing genera: *Clostridiaceae*, *Staphylococcaceae*; ↓ *Helicobacteraceae* and *Campylobacteraceae*	NR	↑ SCFAs (butyrate, propionate); ↓ IL-1β, IL-6, TNF-α; ↓ goblet cells, mucus secretion (FPLP and FPLE more effective for inflammation) (FPP more effective for SCFA production)	Supportive microbial-related; SCFAs	Chen et al. 2026 [92]
Platycodon grandiflorum polysaccharides (PGP) + saponins (PGS) PGP: low and high dose: 200 and 400; PGS: 45; PGP 200 + PGS 45; PGP 400 + PGS 45 mg/kg; by gavage × 30 days	Cigarette and sawdust smoke-induced COPD (Sprague-Dawley rats)	↑ *Lachnospiraceae_NK4A136_group*; ↓ *Allobaculum*, *Clostridia_UCG-014_unclassified*	NR	↓ CCL20, IFN-γ, TNF-α; ↓ neutrophils, lymphocytes, and macrophages in BALF; improve lung and intestine histopathology; ↑ MUC2, ZO-1, ki67 in intestine; ↓ Hyp, NO, TLR4/NF-kB in lung (Combination more effective than monotherapy)	Supportive microbial-related	Xu et al. 2025 [93]
10%, 20%, and 30% PGP	in vitro anaerobic microbiota culture	NR	NR	↑ metabolism of platycodin D and platycodin D3		
20% Cellulose/20% Pectin orally × 3 weeks	Cigarette smoke-induced emphysema (C57BL/6 mice)	↑ *Verrucomicrobia* phyla (genus *Akkermansia*), *Alphaproteobacteria* class (*Rhodospirillaceae* family) (cellulose) ↑ *Bacteroidetes*; strongest SCFA-associated pattern; ↓ *Lactobacillaceae*, *Defluviitaleaceae* (pectin)	NR	↑ SCFAs (acetate, propionate, butyrate), bile acids (CA, CDCA, UDCA), sphingolipids; ↓ macrophage and neutrophil, IFN-γ (N.S.), IL-6 (N.S.) in BALF; ↓ IFN-γ, IL-18, TNF-α in lung tissue; ↓ alveolar destruction and airspace enlargement (pectin more effective)	Supportive microbial-related; SCFAs	Jang et al. 2021 [75]
Polyphenols						
Reineckea carnea (RCK) (water extract) (Polyphenol) low, medium, high dose: 1.5, 3, and 6 g/kg, respectively, by gavage × 2 weeks	Cigarette smoke-induced COPD (Kunming mice)	↑ anamorph phylum; ↓ thick-walled phylum; ↓ *Firmicutes*/*Bacteroidetes* ratio	NR	↓ IL-6, IL-8, TNF-α, NF-κB; ↑ CAT, GSH-PX, SOD and ↓ ROS, MDA; ↓ alveolar dilation, alveolar wall destruction, bronchial wall thickening, inflammatory cell infiltration (dose-dependent)	NF-κB pathway regulation with microbial-related association	Chen et al. 2025 [94]
Acute lung injury						
Dietary fibers						
High-fiber diet (HFD) orally × 3 weeks	HALI (C57BL/6J mice)	↑ acetate-producing bacteria, *Bacteroides acidifaciens*; ↓ *Firmicutes*/*Bacteroidetes* ratio	NR	↑ survival; ↓ TNF-α, IL-6, KC, MDA in BALF; ↓ neutrophil, MPO-positive cells, H_2_O_2_ contents, NFκB, apoptosis in lung; ↓ wet/dry ratio of lung	Anti-inflammation with microbial-related association	Chu et al. 2022 [95]
Partially hydrolyzed guar gum (PHGG) (Fiber) pretreatment 500 mg/kg orally × 4 weeks	H1N1 influenza virus-induced ALI (C57BL/6J mice)	↑ *Clostridia*	NR	↑ SCFAs (acetic, propionic, butyric acid); ↓ IFN-γ, IL-1β, IL-6, IL-10, TNF- α; ↓ intestinal atrophy; ↑ NK-cells and Treg in spleen	Supportive microbial-related; SCFAs	Kato et al. 2023 [96]
Feruloylated oligosaccharides pretreatment 200 or 400 mg/kg orally × 14 days	H1N1 influenza virus-induced ALI (C57BL/6J mice)	↓ *Ruminococcus* (associated with allergies, eczema, asthma)	NR	↑ survival; ↓ IFN-α, IFN-β, IL-6, IL-10, IL-23 in the lung; ↓ RIG-I/MAVS/TRAF3 pathway, NFκB (dose-dependent)	Anti-inflammation and antiviral effects with microbial-related association	Deng et al. 2024 [97]
Serially diluted FOs	in vitro mixed with virus	NR	NR	↓ viral markers (NP, M2); moderate hemagglutination inhibition and neuraminidase inhibition (dose-dependent)		
*Houttuynia cordata* Polysaccharides (HCP) 40 mg/kg by gavage × 4 days	H1N1 influenza virus-induced ALI (BALB/c mice)	NR ↑ *Lachnospiraceae*; ↓ *Bacteroides* (microbiota data only in FMT group)	NR	↑ survival; ↑ acetate; ↓ percentage of total and migrated Th17 cells; improve inflammation in lung and intestinal histopathology	Direct microbial-related; confirm with FMT after broad-spectrum antibiotics-induced microbiota depletion	Shi et al. 2022 [98]
*Houttuynia cordata* Polysaccharides (HCP) (water extract) 20 or 40 mg/kg by gavage × 4 days	H1N1 influenza virus-induced ALI (BALB/c mice)	NR	NR	↓ Th17, IL-17A, CCR6+, Th17 migration, lung CCL20; ↑ Treg, IL-10, CCR6+ Treg migration; improve inflammation in lung and intestinal histopathology (uncertain dose-dependent)	Mucosal immune mechanism Uncertain microbial-related	Shi et al. 2020 [99]
100 µg/mL × 72 h	in vitro primary naïve CD4^+^ T cells	NR	NR	↓ Th17 differentiation (lower phospho-STAT3); ↑ Treg differentiation (higher phospho-STAT5)		
*Houttuynia cordata* Polysaccharides (HCP) 40 mg/kg by gavage × 4 days	H1N1 influenza virus infected (BALB/c mice)	↓ *Vibrio*, *Bacillus*	NR	↓ TLRs, IL-1β; ↑ IL-10; ↓ HIF-1α, mucosubstances in goblet cells; ↑ ZO-1 in intestine	Supportive microbial-related	Chen et al. 2019 [100]
*Houttuynia cordata* Polysaccharides (HCP) 20 or 40 mg/kg by gavage × 4 days	H1N1 influenza virus-induced ALI (BALB/c mice)	NR	NR	↑ survival; ↓ viral replication; ↓ TNF-α, IL-6, IFN- α, TLR4, p-NFκB p65 in lung; ↑ IgA, ZO-1 and ↓ goblet cells in intestine (dose-dependent)	Mucosal barrier restoration Uncertain microbial-related	Zhu et al. 2018 [101]
*Houttuynia cordata* Polysaccharides and Flavonoids (HCP and HCF) HCP 80; HCF 100; HCP 40 + HCF 100; HCP 80 + HCF 100; HCP 80 + HCF 200 mg/kg by gavage × 7 days	H1N1 influenza virus-induced ALI (BALB/c mice)	↑ *Bacteroidetes*; ↓ *Firmicutes, Proteobacteria*	NR	↑ survival; ↓ MCP-1, IL-8, TNF-α, IL-6, IL-1β; ↑ IL-10; ↓ lung apoptosis, macrophage and neutrophil infiltration, viral titers, lung index, injury area, and histologic inflammation (combination more effective than monotherapy) (HCF more effective for antiviral) (HCP more effective for physical barrier)	Supportive microbial-related	Ling et al. 2023 [102]
*Astragalus* polysaccharides (APS) pretreatment 200 mg/kg by gavage × 14 days	LPS-induced ALI (C57BL/6 mice)	↑ SCFA-producing genera: *Oscillospira*, *Akkermansia*, *Coprococcus*	NR	↑ SCFAs (butyrate, propionate); transition of macrophages from pro-inflammatory to anti-inflammatory phenotype; improve lung histopathology, ↓ neutrophil infiltration	Supportive microbial-related: SCFAs	Ming et al. 2022 [103]
*Lycium ruthenicum* Murr. Polysaccharide pretreatment low and high dose: 100 and 400 mg/kg, respectively, by gavage × 7 days	LPS-induced ALI (C57BL/6J mice)	↑ *Bacteroidetes*; ↓ *Escherichia-Shigella*	NR	↓ TNF-α, IL-1β, IL-6; ↑ IL-10; ↓ total cells, macrophages, neutrophils, lymphocytes in serum and BALF; ↓ alveolar collapse (dose-dependent)	Anti-inflammation with microbial-related association; SCFAs/GPR/HDAC	Lu et al. 2025 [104]
50, 100, 150, 200 µg/mL × 24 h	in vitro A549 cells	NR	NR	↑ %viable A549 cells; ↓ TNF-α, IL-1β, IL-6; ↑ IL-10 (dose-dependent)		
Glycyrrhiza uralensis Polysaccharide (GCP-2) (aqueous extract) pretreatment low and high dose: 300 and 600 mg/kg, respec-tively by gavage × 12 days	LPS-induced ALI (C57BL/6 mice)	↑ *Bacteroidetes*, *Lactobacillus*, *Bifidobacteria*; ↓ *Firmicutes*, *Proteobacteria*	NR	↓ IL-1β, IL-6, TNF-α; ↑ TJ proteins (ZO-1, occludin, claudin) in lung; ↓ TLR4/NFκB pathway; improve lung and intestine histopathology (dose-dependent)	Supportive microbial-related	Yu et al. 2025 [105]
*Anemarrhena asphodeloides* (Polysaccharide) 400 mg/kg orally × 14 days	LTA-induced pneumonia BALB/c mice	↑ *Firmicutes*; ↓ *Bacteroidetes*, *Campylobacteria*	NR	↓ symptoms; TNF-α, IL-10; ↑ MUC-2, TJ proteins (ZO-1, occludin, claudin-1) in intestine; improve lung and intestine histopathology	Supportive microbial-related	Wen et al. 2024 [106]
Polyphenols						
Black tea infusion (TI); Ethanol-soluble fraction (ES), Ethanol-precipitate fraction (EP) (Polyphenol) 7.5, 15, and 22.5 mg/mL as drinking fluid orally × 4 weeks	PM-induced ALI (BALB/c mice)	↑ *Lachnospiraceae* (TI, ES, EP)	NR	↓ IL-6, CXCL1, CXCL2, CXCL15 in BALF; ↓ IL-6 and TNF-α in serum, ↓ NF-κB/STAT3 in lung (EP most effective) (dose dependent)	Direct microbial-related; confirm with FMT	Zhao et al. 2021 [107]
Epigallocatechin-3-gallate (EGCG) (Polyphenol) pretreatment 10 or 20 mg/kg by gavage × 7 days	LPS-induced ALI (C57BL/6J mice)	↑ *Akkermansia muciniphila*	NR	↑ SCFAs (acetate, proprionate, butyrate); ↓ JAK2/STAT3 pathway; ↓ IL-1β, IL-6, TNF-α in plasma and BALF; ↓ inflammatory cell infiltration in lung; ↑ ZO-1, occludin (dose-dependent)	Direct microbial-related; SCFAs confirm with FMT and SCFAs supplement	Fan et al. 2026 [108]
Tannic acid (TA) (Polyphenol) 200 or 400 mg/kg as diet orally × 21 days	Salmonella-induced ALI (White-feathered broilers)	↑ *Bacteroides fragilis*; ↓ *Pelomonas*, *Escherichia-Shigella*, *Ralstonia*	NR	↓ IL-4 IL-6, IFN-γ; ↓ serum LPS; ↓ MPO in lungs; improve lung and intestinal histopathology; inhibit M1 macrophage polarization in the lungs (uncertain dose-dependent)	Supportive microbial-related; intestinal barrier restoration	Wu et al. 2025 [109]
Fermented black barley (Hordeum distichum L.) (Polyphenol) 1 mL/100 g BW via gavage × 9 weeks	Cooking oil fume-induced ALI (ICR mice)	↑ *Firmicutes*, *Lactobacillus*; ↓ *Bacteroidetes*	NR	↑ SOD activity in lung; ↓ wet/dry ratio of lung; ↑ cilium assembly and the cilium axoneme genes	Upregulation of genes involved in cilium assembly and cilium axoneme with microbial-related association	Ding et al. 2020 [110]
Baicalein (BAI) (Flavonoid) pretreatment 30 or 60 mg/kg orally × 14 days	SEB-induced ARDS/ALI (C57BL/6J mice)	↑ SCFA-producing family: *Prevotellaceae*	NR	↑ SCFAs (acetic, propionic, butyric, valeric acid); ↓ TLR4/MyD88; ↑ ZO-1, claudin, occludin-1 in lung tissue (dose-dependent)	Direct microbial-related; confirm with FMT after broad-spectrum antibiotics-induced microbiota depletion	Hu et al. 2024 [111]
Resveratrol (RES) (Polyphenol) pretreatment 100 mg/kg by gavage × 2 doses at 24 h and 90 min before SEB	SEB induced ARDS/ALI (C3H/HeJ mice)	↑ *Actinobacteria*, *Tenericutes*, *Lactobacillus reuteri*	↑ *Actinobacteria*, *Tenericutes*, *Lactobacillus reuteri*	↑ survival; ↑ SCFA (iso-butyric acids); ↓ IFN-γ, IL-1β, IL-17; ↑ IL-10; ↓ mononuclear cells, cytotoxic CD8+ T infiltration in lungs	Immunomodulatory effect and direct microbial-related confirm with FMT	Alghetaa et al. 2021 [112]

ACQ, asthma control questionnaire; AHR, airway hyperresponsiveness; ALI, acute lung injury; ARDS, acute respiratory distress syndrome; BALF, bronchoalveolar lavage fluid, BW, body weight; CA, cholic acid; CAT, catalase; CDCA, chenodeoxycholic acid; CCL, chemokine C-C motif ligand; COPD, chronic obstructive pulmonary disease; CXCL, chemokine C-X-C motif ligand; dT, delay time; FMT, fecal microbiota transplantation; FOS, fructooligosaccharide; GOS, galactooligosaccharide; GSH-PX, glutathione peroxidase; h, hour; HALI, hyperoxia-induced acute lung injury; HDAC9, histone deacetylase 9; HDM, house dust mite; HIF, hypoxia inducible factor; Hyp, hydroxyproline; IFN, interferon; IL, interleukin; i.p., intraperitoneal; JAK, janus kinase; LTA, lipoteichoic acid; KC, keratinocyte-derived chemokine; MCP, monocyte chemoattractant protein, MDA, malondialdehyde; MPO, myeloperoxidase; MUC, mucin; MV, mean volume; MyD, myeloid differentiation primary response; M2, matrix protein 2; N, number of participants; NK, natural killer; NP, nucleoprotein; NR, not report; 2-NT, 2-naphthol; OHP, 1-hydroxypyrene; N.S., not significant; OVA, ovalbumin; PI3K/Akt, phosphoinositide 3-kinase/protein kinase B; PM, particulate matter; RIG, retinoic-acid inducible gene; ROS, reactive oxygen species; SCFA, short chain fatty acid; SEB, *Staphylococcal* Enterotoxin B; SOD, superoxide dismutase; sRaw, specific airway resistance; STAT, signal transducer and activator of transcription; Th, T helper cell; TJ, tight junction; TNF, tumor necrosis factor; TLR, Toll-like receptors; Treg, regulatory T cell; TRP, transient receptor potential; UDCA, ursodeoxycholic acid; wt:v, weight per volume; ZO, zonula occluden. ↑ means increase and ↓ means decrease.

## Data Availability

No new data were created or analyzed in this study. Data sharing is not applicable to this article.

## Abbreviations

The following abbreviations are used in this manuscript:

AhR	aryl hydrocarbon receptor
ARDS	acute respiratory distress syndrome
CCL	chemokine C-C motif ligand
CCL25–CCR9	the chemokine C-C motif ligand 25- chemokine C-C motif receptor 9
CCR	chemokine C-C motif receptor
COPD	chronic obstructive pulmonary disease
CXCL1	C-X-C motif chemokine ligand
CX3CR1	C-X3-C motif chemokine receptor 1
DAT	desaminotyrosine
EGCG	epigallocatechin-3-gallate
FFAR	free fatty acid receptor
Foxp3	Forkhead Box Protein P3
GALT	gut-associated lymphoid tissue
GPR	G protein-coupled receptor
HCAR	hydroxycarboxylic acid receptor
HDAC	histone deacetylase
HCP	*Houttuynia cordata* polysaccharides
IgA	immunoglobulin A
IgE	immunoglobulin E
IL	interleukin
ILC	innate lymphoid cells
JAK	janus kinase
LPS	lipopolysaccharide
Ly6c	lymphocyte antigen 6 complex, locus C1
MAMP	microbe-associated molecular pattern
MyD	myeloid differentiation primary response
NF-κB	nuclear factor-κB
NLR	NOD-like receptor
PA	polyamine
PGN	peptidoglycan
PHGG	partially hydrolyzed guar gum
PRR	pattern-recognition receptor
SCFA	short-chain fatty acid
SFB	segmented filamentous bacteria
spp.	species
STAT	signal transducer and activator of transcription
TGF-β	transforming growth factor β
Th	T helper
TLR	toll-like receptor
TNF-α	tumor necrosis factor-α
Treg	regulatory T cells
